# Clinical and Etiological Aspects of Gynecomastia in Adult Males: A Multicenter Study

**DOI:** 10.1155/2018/8364824

**Published:** 2018-05-29

**Authors:** Pablo René Costanzo, Néstor Antonio Pacenza, Sergio Mario Aszpis, Sebastián Matías Suárez, Uriel Marcelo Pragier, Jorge Guillermo Stewart Usher, Miguel Vásquez Cayoja, Sergio Iturrieta, Silvia Elisa Gottlieb, Rodolfo Alberto Rey, Pablo Knoblovits

**Affiliations:** ^1^Servicio de Endocrinología, Metabolismo y Medicina Nuclear, Hospital Italiano, Buenos Aires, Argentina; ^2^Servicio de Endocrinología y Metabolismo, Unidad Asistencial “Dr. César Milstein”, Buenos Aires, Argentina; ^3^Centro de Endocrinología y Diabetes “Dr. Raúl Gutman”, Buenos Aires, Argentina; ^4^División Endocrinología, Hospital Durand, Buenos Aires, Argentina; ^5^Servicio de Endocrinología, Complejo Médico Churruca-Visca, Buenos Aires, Argentina; ^6^Consultorio de Endocrinología, Centro Médico Haedo, Buenos Aires, Argentina; ^7^Servicio de Endocrinología, Hospital Ramos Mejía, Buenos Aires, Argentina; ^8^Centro de Investigaciones Endocrinológicas “Dr. César Bergadá” (CEDIE), CONICET – FEI, División de Endocrinología, Hospital de Niños Ricardo Gutiérrez, Buenos Aires, Argentina

## Abstract

**Objectives:**

To evaluate the characteristics of presentation, biochemical profile, and etiology of gynecomastia in adults.

**Methods:**

Medical records of 237 men aged 18-85 years with gynecomastia were evaluated.

**Results:**

Highest prevalence of gynecomastia was observed between 21 and 30 years (n = 74; 31.2%). The most common presenting complaints were aesthetic concerns (62.8%) and breast pain (51.2%). 25.3% of the subjects had a history of pubertal gynecomastia. 56.5% had bilateral gynecomastia. 39.9% were overweight and 22.8% were obese. The etiology could not be identified in 45.1% of the cases; the most frequent identified causes were anabolic steroids consumption (13.9%), hypogonadism (11.1%), and use of pharmaceutical drugs (7.8%). Patients with bilateral gynecomastia had a longer history of disease, higher BMI, and lower testosterone levels.

**Conclusions:**

Patients with gynecomastia presented more often with aesthetic concerns and secondarily with breast pain. The most frequent final diagnosis was idiopathic gynecomastia, whereas the most frequent identified etiologies were anabolic steroids consumption, hypogonadism, and use of pharmaceutical drugs. Despite the low frequency of etiologies such as thyroid dysfunction or adrenal carcinoma, we emphasize the importance of a thorough assessment of the patient, as gynecomastia may be the tip of the iceberg for the diagnosis of treatable diseases.

## 1. Introduction

Gynecomastia, defined as the benign proliferation of breast glands in males, is a common complaint that produces anxiety and discomfort and it may be the expression of a clinically relevant disease [1, 2].

Gynecomastia may be diagnosed on routine clinical examination or patients may present with complaints of a retroareolar nodule. This condition may occur sporadically or in a familial setting, and it may be unilateral or bilateral, painful or painless, of acute onset or progressive growth [3–5].

The hormones involved in breast tissue physiology may be stimulatory (as estradiol and progesterone) or inhibitory (as testosterone), acting directly through their specific receptors at this level [6, 7]. Receptors for insulin-like growth factor 1 (IGF-1), IGF-2 [8], luteinizing hormone, and human chorionic gonadotropin have also been detected in breast tissue [7, 8]. Estrogens and progesterone apparently require the presence of growth hormone and IGF-1 to exert their stimulatory action on the breast [9]. Hyperprolactinemia may indirectly cause gynecomastia by suppressing gonadotropin-releasing hormone (GnRH) release, resulting in central hypogonadism, although prolactin receptors have also been detected in benign and malignant breast tissue. At breast level, prolactin might modulate progesterone and androgen receptors expression (increasing the former and reducing the latter) [10]. Furthermore, prolactin stimulates epithelial cell proliferation only in the presence of estrogen and enhances lobuloalveolar differentiation only with concomitant progesterone [7].

Gynecomastia may result from an excess of estrogens (obesity, tumors, and exogenous sources) [11], androgen deficiency (hypogonadism), hormone resistance [12], or altered ratio of estrogens to androgens (refeeding, liver disease, and renal failure) [13].

It may also be a physiological phenomenon in different stages of life, such as in the newborn, during pubertal development [14], and in the elderly [15, 16], or it may be a pathological condition caused by drugs of abuse [17, 18], systemic disease [17, 19], endocrine disorders, tumors, and medications [17]. Even though gynecomastia is a common condition, e.g., palpable breast tissue may be detected in one- to two-thirds of adult males, while autopsy data suggest a prevalence of 40-55% [19–23], the relative prevalence of the various etiologies has only been investigated in a few series, most of them with a low number of patients. Further studies are required in a larger number of subjects to evaluate the etiology and characteristics of this disease.

The aims of our study were to evaluate the etiology and the clinical presentation of gynecomastia and the biochemical profile in a group of adult men seeking specialized endocrine care.

## 2. Material and Methods

All the medical records of males aged >18 years who presented with gynecomastia or were diagnosed with gynecomastia when presenting with other complaints from May 2004 to June 2014 were evaluated in a cross-sectional, analytical, retrospective, multicenter study. The sites where patients were seen are located in Buenos Aires and are attended by a population with middle to high cultural and socioeconomic status.

Inclusion criteria for medical records were as follows:

(i) Data on the following clinical factors: breast pain, duration, presence of galactorrhea, habits (alcohol, drugs of abuse, and anabolic drugs), history of pubertal gynecomastia, medical history, and use of medication. The consumption of marijuana and other drugs was self-reported.

(ii) Hormonal laboratory assessment: total testosterone (TT), estradiol (E2), luteinizing hormone (LH), follicle-stimulating hormone (FSH), prolactin (PRL), and thyroid-stimulating hormone (TSH).

(iii) Imaging confirming diagnosis (mammography and/or ultrasound).

In all patients, gynecomastia was confirmed by ultrasound and/or mammography.

The following data were collected from physical examination: weight and height; body mass index (BMI) was calculated using the formula: weight (kg)/height (m)^2^. A BMI 18.5-25 kg/m^2^ was considered normal weight, a BMI ≥25 and <30 kg/m^2^ was considered overweight and a BMI ≥30 kg/m^2^ was considered obese. A breast examination was performed: the patient lies flat on his back with his hands clasped beneath his head. Using the separated thumb and forefinger, the examiner slowly brings the fingers together from either side of the breast. True glandular breast tissue (gynecomastia) can be distinguished from fatty breasts by comparing subareolar tissue with adjacent subcutaneous fat (such as that in the anterior axillary fold). Gynecomastia is felt as symmetric, firm glandular tissue under the nipple. Testicular examination was performed by palpation of both testes and measurement of their volume by Prader orchidometer. Given the low rate of testicular tumors that may be associated with gynecomastia, palpation was performed in all patients, while testicular ultrasound was not performed as routine, but only requested when warranted by palpation findings or in cases of hyperestrogenism of unknown etiology.

Routine and hormonal laboratory data were analyzed: blood glucose, lipid profile, liver function tests, creatinine, blood count, TT, E2, LH, FSH, PRL, and TSH. Oncologic markers had also been ordered in most patients: alpha-fetoprotein, *β*-subunit of human chorionic gonadotropin, and carcinoembryonic antigen.

Hormonal measurements were performed using the assay available at the site where the patient was seen (see Supplementary Information (available here)). Blood samples were taken at 8:00 am after 12-hour nocturnal fasting. For biochemical abnormalities, reference values of each site were considered.

Hypogonadism was defined by TT levels <3.0 ng/mL, confirmed by repeat TT measurement [24], hyperprolactinemia by PRL levels >20 ng/mL, and hyperestrogenism by E2 levels >60 pg/mL.

Regarding age we divided the population into older and younger than 40 years because (1) in longitudinal studies it was shown that testosterone begins to gradually fall after this age, (2) the median age of the population in this study is closer to 40 years, and finally (3) the number of patients between decades 41-50, 51-60, 61-70, 71-80, and >80 years is more homogeneous than decades < 40 years.

### 2.1. Statistical Analysis

Data were analyzed using Instat Statistical Software (GraphPad, version 3.01). Differences in the characteristics between patients with unilateral or bilateral gynecomastia were compared with a two-sample *t*-test (parametric) or Mann–Whitney U test (not parametric) for continuous variables. Data are presented as the mean ± SD or median and range as appropriate. All *p* values quoted are two-sided, and values below 0.05 were regarded as statistically significant.

## 3. Results

The medical records of 435 males with a diagnosis of gynecomastia were reviewed and 237 of these records met the inclusion criteria. The enrollment was performed as follows: Department of Endocrinology, Metabolism and Nuclear Medicine, Hospital Italiano n = 145; Endocrinology Division, Hospital Durand n = 44; Department of Endocrinology and Metabolism, Unidad Asistencial “Dr. César Milstein” n = 18; Department of Endocrinology, Centro Médico Haedo n = 17; Department of Endocrinology, Complejo Médico Churruca-Visca n = 9; Department of Endocrinology, Hospital Ramos Mejía n = 4.

Diagnosis was documented only by ultrasound in 56.2% of patients, only by mammography in 16.4% of patients and by both in 27.4% of patients.

**Figure 1:**
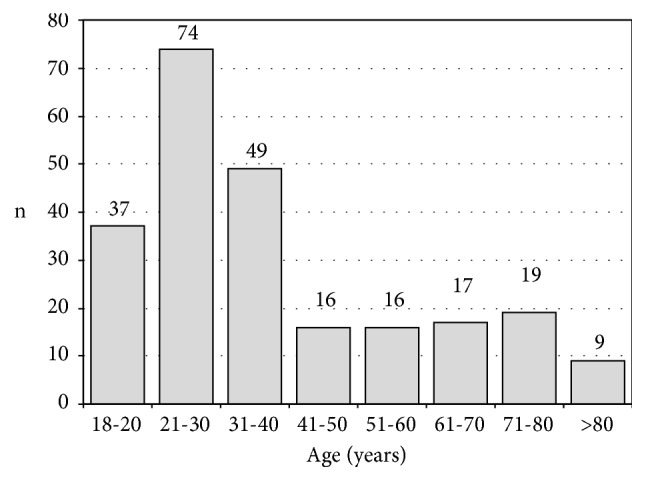
Age distribution of patients presenting with gynecomastia (n = 237).

The median age at the time of the first visit was 32 years (range: 18 to 85 years).  Figure 1 shows the age distribution of patients.

Most patients presented spontaneously at the endocrinology office (72.5%); the rest were referred by other specialists. Main complaints included esthetic concerns in 62.8% of patients and pain in 51.2% (20.2% for both complaints). Gynecomastia was incidentally found on physical examination in 3.9% of patients seeking medical attention for other reasons.

At the clinical interview, 25.3% of the study subjects reported a history of pubertal gynecomastia. The duration of disease before seeking specialized endocrine care was highly variable, ranging from 1 month to 40 years (median: 1 year).

On physical examination, 134 patients (56.5%) had bilateral and 103 (43.5%) unilateral gynecomastia (left in 54.4% and right in 45.6%). The 39.9% of the study subjects were overweight and 22.8% had obesity. The mean BMI was 27.0 ± 4.5 kg/m^2^. In 28.7% of patients, breast pain was found on physical examination. One patient (with macroprolactinoma) reported spontaneous nipple discharge and in 3 patients, nipple discharge was found on physical examination. Of 156 with gonadal examination description, 126 (80.8%) had normal gonadal volume, 19 (12.2%) had bilateral hypotrophy, 7 (4.5%) had unilateral hypotrophy, and 4 (2.5%) had unilateral absence of the testicle (2 with hypotrophic single testicle and 2 with trophic single testicle). Absence of the testicle was confirmed by testicular ultrasound and abdominal MRI in all cases.

As regards the analysis of etiologies, 134 causes were identified in 127 patients; in 7 cases 2 causes coexisted (Table 1). Among the causes that were identified, consumption of anabolic steroids was the most common (13.9%). Hypogonadism was found in 11.1% of patients, hyperprolactinemia in 5.7%, and hyperestrogenism in 0.4%. Of 27 patients with hypogonadism, 20 were hypergonadotropic (9 patients with Klinefelter's Syndrome) and 7 were hyponormogonadotropic. Of the 14 patients with hyperprolactinemia, 6 had prolactinoma, and all others were considered to have idiopathic hyperprolactinemia. Drug-induced hyperprolactinemia was included within pharmacological causes. The drugs involved were finasteride (5 cases), antiretrovirals (4), spironolactone (4), bicalutamide (1), dutasteride (1), pimozide (1), sulpiride (1), flutamide (1), and LH-RH analog (1). No elevated tumor markers were found in any of the cases. In 110 cases (45.1%), it was not possible to establish an etiology and gynecomastia was classified as idiopathic.

An analysis of etiologies according to age was performed. The etiologies and hormonal values in <40 and >40 years are described in Tables 2 and 3, respectively.

A certain etiology was more frequently identified in young adults: anabolic consumption in patients with a mean (± SD) age of 30.3 ± 7.2 years, persistent puberty: 24.4 ± 10.0 years, hyperprolactinemia: 34.9 ± 14.1 years, and marijuana consumption: 27.3 ± 8.0 years. Conversely, gynecomastia secondary to pharmacological drugs use was more frequent in the elderly: 84.2% were older than 40 years, with a mean age of 62.5 ± 20.6 years. The mean age of consultation for gynecomastia in patients with hypogonadism was 48.6 ± 23.1 years, yet the subgroup of patients with Klinefelter's Syndrome (KS) were younger: 23.9 ± 6.9 years old.

Patients with bilateral gynecomastia had a longer time of evolution as compared to those with unilateral disease: 3.4 ± 5.6 versus 1.4 ± 1.9 years (p = 0.0001); higher BMI: 27.8 ± 4.7 versus 25.7 ± 4.0 kg/m^2^ (p = 0.005); and lower TT levels: 4.6 ± 2.1 versus 5.3 ± 2.0 ng/mL (p = 0.008), respectively.

## 4. Discussion

In this series of patients presenting with gynecomastia, etiologies of gynecomastia were found in 54.9% of cases. Among the causes detected, the use of anabolic steroids and persistent pubertal gynecomastia were the most common in the young population, while hypogonadism and the use of drugs were the most common in elderly patients. The most common complaints were esthetic concerns and breast pain. Detection of galactorrhea was rare, gonadal examination was normal in most patients, and 62.7% were overweight or obese. As regards clinical presentation, our data show that unilateral gynecomastia (there were no differences between right and left occurrences) is almost as frequent as bilateral gynecomastia. Anyway, it is important to remember that, from a pathologic point of view, there is usually bilateral involvement [23–25]. Slightly over half of patients presented with bilateral gynecomastia, and when compared with cases of unilateral gynecomastia these patients demonstrated a longer duration of disease, higher BMI, and lower TT levels.

Gynecomastia is a common entity that may be brought to the attention of the physician by the patient himself or it may be found on a clinical examination performed for other health problems. This difference in the way it is detected (incidental finding or main complaint) determines and places a bias on the forms of presentation and the various etiologies reported by different published case series [26, 27]. Furthermore, the different methods of assessment at each site certainly determine the higher or lower reported frequency of idiopathic gynecomastia and of each probable etiology.

Our analysis, though retrospective, included only cases studied by a complete clinical, biochemical, and imaging assessment, as suggested by various authors [2, 8, 16, 17]. Almost all patients presented directly for evaluation of gynecomastia and in only 3.9% of the cases included in this series this condition was discovered in patients who presented with other complaints. The fact that 72.5% of patients initially sought advice from an endocrinologist is likely to be biased by our own methodology, as only cases with complete diagnostic assessments were eligible for inclusion and such requirement is more likely to be met in the endocrinology setting than in other specialties.

As gynecomastia is a chronic—often asymptomatic—process, patients do not immediately seek medical attention. In these cases, patients are referred to the specialist long after the onset of signs and symptoms. Instead, in cases with a recent onset of symptoms, which may be pain or discomfort, medical attention is sought in the short term. The duration of gynecomastia at the time of seeking medical attention in our population was highly variable, ranging from 1 month to 40 years.

One-fourth of the patients had a history of pubertal gynecomastia but in only 6.2% of cases persistence of this condition was the main complaint. The frequency of pubertal gynecomastia reported in different studies is higher than that reported as history in our population [15, 21, 28]. Probably, the prevalence of the history of pubertal gynecomastia in our study was not higher because it was not as clinically relevant as to be reported by patients. Furthermore, our criterion of including only patients older than 18 also creates a bias regarding the prevalence of this type of gynecomastia.

Patients with bilateral gynecomastia had longer duration of the condition, higher BMI, and lower TT levels than patients with unilateral gynecomastia. No differences occurred in plasma E2 levels between unilateral and bilateral conditions, as it did occur in TT levels. It could be assumed, in relation to pathophysiology, that patients with bilateral presence of breast tissue have higher E2 levels locally, though not peripherally, or that the longer duration of the condition permitted chronic stimulation, which resulted in the bilateral enlargement. Another possibility would be that patients with lower testosterone levels might have a greater magnitude of gynecomastia because of the lower inhibitory effect of this hormone on breast tissue.

The proportion of patients with overweight and obesity in our population was slightly higher than that reported in a study conducted in an Argentine population of men aged between 35 and 64 years (39.9% and 22.8% versus 34.8% and 14.8%, respectively) [29]. These observations would support the concept of obesity as predisposing or “etiologic” factor; however, we are aware that a larger sample is required to draw conclusions about the specific influence of overweight and obesity on gynecomastia.

As reported in other series, idiopathic gynecomastia was the most common finding (45.1% of cases), although in our study the percentage was lower than that reported in other studies (58-61%) [27, 30–32]. The fact that all patients were evaluated in endocrinology departments and that biochemical and imaging tests were part of the inclusion criteria may account for the identification of more causes. Nevertheless, we have to mention also that almost 50% (45.5%) of the men initially identified were excluded, which shows that half of the patients with gynecomastia are not completely studied, at least in our institutions.

Among the causes identified, the most prevalent was the use of anabolic steroids (13.9%). The rate of occurrence of the various etiologies is related to the age of the population: use of anabolic steroids in younger patients and use of pharmaceutical drugs and hypogonadism in older ones. When dividing the population into older and younger than 40 years, as shown in Tables 2 and 3, the rate of occurrence of the various etiologies changes, with no significant variation in the rate of the idiopathic etiology. Among detectable causes in subjects younger than 40 years, the most prevalent were the use of anabolic steroids, persistent pubertal gynecomastia, hyperprolactinemia, hypogonadism and the use of marijuana. These five causes constitute 89.3% of identifiable causes in this group. In the group of men older than 40 years, hypogonadism and the use of pharmaceutical drugs were the most common causes, accounting for 72.1% of secondary causes in this age group.

Despite the low frequency of etiologies such as thyroid dysfunction or adrenal carcinoma, we emphasize the importance of a thorough assessment of the patient, as gynecomastia may be the tip of the iceberg for the diagnosis of potentially treatable diseases. In nine cases (3.8%), gynecomastia was associated with KS. The prevalence of KS in this series is relatively low because only those cases in which gynecomastia was the main complaint were included. In a previous study where 54 patients with KS older than 18 years were evaluated, we found a prevalence of gynecomastia of 31.3% [33]. Furthermore, patients with KS are likely to seek medical attention for gynecomastia before the age of 18 (at a younger age than that of our study population). In addition to the KS cases, 11 patients had hypogonadism with elevated gonadotropin levels. All of them had normal karyotype and/or testicular volume inconsistent with KS.

The etiology of gynecomastia has been evaluated in a reduced number of published series. In 53 young patients, the etiology was found to be idiopathic in 58% of cases, while secondary causes included hypogonadism (25%), hyperprolactinemia (9%), chronic liver disease (4%), and drug-induced disease (4%) [31]. In another retrospective study of 87 patients, idiopathic gynecomastia was detected in 61% of patients, while this disease was induced by drugs in 21%, by liver or kidney disease in 16% and hyperthyroidism in 2% [27]. Mieritz et al. found 57% of “unexplained gynecomastia” and 15.2% of patients had testosterone deficiency [32].

All patients had imaging studies (ultrasound and/or mammography) as this was an inclusion criterion. Both ultrasound and mammography may also help to differentiate between lipomastia and gynecomastia if doubts arise during physical examination. Mammography is the study of choice when breast cancer is suspected, as it has a negative predictive value above 90%, and over 90% sensitivity and specificity for differentiating between benign and malignant disease in the breast [27]. Testicular ultrasound was ordered when abnormal findings were detected on palpation of the testes or in cases of hyperestrogenism of unknown etiology. No testicular pathology was detected on ultrasound when performed.

In conclusion, the analysis of these results suggests that although there are a large proportion of idiopathic cases, gynecomastia may be the expression of a relevant underlying clinical condition. This highlights the need for an adequate and complete clinical, biochemical, and imaging assessment of these patients.

## Figures and Tables

**Table 1:** Causes of gynecomastia in 237 patients (n = 244).

Causes	N	%	TT (ng/mL)	Prolactin (ng/mL)	TSH (uIU/mL)	LH (mIU/mL)	FSH (mIU/mL)	E2 (pg/mL)
Anabolic steroids	34	13.9	5.6 ± 1.3	13.7 ± 8.5	1.9 ± 0.8	4.0 ± 2.9	3.6 ± 1.9	30.3 ± 14.7
Hypogonadism	27	11.0	2.1 ± 1.1	13.9 ± 9.3	2.4 ± 0.6	19.2 ± 14.3	31.0 ± 23.2	29.9 ± 16.3
Pharmaceutical drugs	19	7.8	4.8 ± 2.0	24.1 ± 34.1	1.9 ± 0.8	11.5 ± 12.5	17.1 ± 21.3	35.9 ± 14.7
Persistent puberty	15	6.2	4.8 ± 2.0	13.7 ± 5.7	1.7 ± 1.0	4.8 ± 2.4	4.6 ± 4.3	30.1 ± 10.8
Hyperprolactinemia	14	5.7	4.2 ± 1.9	167.4 ± 335.9	2.8 ± 1.9	6.0 ± 6.2	12.0 ± 19.5	23.1 ± 9.7
Marijuana	8	3.3	8.3 ± 2.7	9.8 ± 3.1	2.5 ± 1.6	4.9 ± 1.6	3.3 ± 2.1	44.0 ± 20.3
Renal Failure	5	2.1	4.2 ± 2.3	16.7 ± 10.6	2.2 ± 1.4	8.9 ± 6.9	13.3 ± 14.9	24.2 ± 7.3
Hyperthyroidism	5	2.1	5.7 ± 2.6	11.0 ± 6.7	0.02 ± 0.02	4.0 ± 2.0	3.7 ± 1.8	36.0 ± 19.0
Chronic liver disease	3	1.2	4.5 ± 2.1	11.2 ± 4.4	1.9 ± 0.3	4.3 ± 0.6	5.6 ± 1.6	44.0 ± 30.8
Dietary phytosteroids	1	0.4	7.6	8.5	0.86	2.4	1.2	48
Hyperestrogenism	1	0.4	4.5	20	2.5	3.6	4	114
Refeeding	1	0.4	12.7	10.4	4.7	4.1	0.9	58
Adrenal carcinoma	1	0.4	2.4					78.1

Idiopathic	110	45.1	5.3 ± 1.8	12.3 ± 4.8	2.1 ± 1.2	4.3 ± 2.3	5.4 ± 5.6	28.4 ± 8.9

**Table 2:** Causes of gynecomastia and hormonal profiles in 160 patients 18-40 years old (n = 165).

Causes	n	%	TT (ng/mL)	Prolactin (ng/mL)	TSH (uIU/mL)	LH (mIU/mL)	FSH (mIU/mL)	E2 (pg/mL)
Anabolic steroids	32	19.4	5.7 ± 1.4	13.9 ± 8.7	1.9 ± 0.8	4.1 ± 3.0	3.5 ± 1.8	30.7 ± 15.0
Persistent puberty	14	8.5	5.0 ± 2.0	14.5 ± 5.1	1.8 ± 1.0	5.1 ± 2.3	4.6 ± 4.4	30.8 ± 10.9
Hyperprolactinemia	11	6.7	5.1 ± 2.0	185.4 ± 375.4	2.9 ± 2.3	7.4 ± 9.9	8.6 ± 13.9	29.8 ± 9.0
Hypogonadism	11	6.7	1.7 ± 1.9	13.0 ± 7.7	2.2 ± 0.4	19.0 ± 14.0	36.4 ± 24.8	30.3 ± 23.1
Marijuana	8	4.8	8.3 ± 2.7	9.8 ± 3.1	2.5 ± 1.6	4.9 ± 1.6	3.3 ± 2.1	44.0 ± 20.3
Pharmaceutical drugs	4	2.4	6.0 ± 0.8	13.9 ± 2.7	2.5 ± 1.0	5.2 ± 2.5	3.2 ± 2.5	31.7 ± 22.8
Hyperthyroidism	3	1.8	5.7 ± 2.6	11.0 ± 6.7	0.02 ± 0.02	4.0 ± 2.0	3.7 ± 1.8	36.0 ± 19.0
Renal failure	2	1.2	5.4 ± 0.8	16.5 ± 14.8	1.4 ± 0.3	5.9 ± 0.5	2.3 ± 0.1	18.5 ± 2.1
Dietary phytosteroids	1	0.6	7.6	8.5	0.86	2.4	1.2	48
Hyperestrogenism	1	0.6	4.5	20	2.5	3.6	4	114
Refeeding	1	0.6	12.7	10.4	4.7	4.1	0.9	58
Chronic liver disease	1	0.6	4.9	8.7	1.9	3.7	4.2	18

Idiopathic	76	46.1	5.7 ± 1.8	12.5 ± 4.6	2.1 ± 1.1	3.9 ± 2.0	3.5 ± 2.2	29.2 ± 9.4

**Table 3:** Causes of gynecomastia and hormonal profiles in 77 patients older than 40 years old (n = 79).

Causes	n	%	TT (ng/mL)	Prolactin (ng/mL)	TSH (uIU/mL)	LH (mIU/mL)	FSH (mIU/mL)	E2 (pg/mL)
Hypogonadism	16	20.3	2.3 ± 0.7	14.4 ± 10.4	2.4 ± 0.7	18.0 ± 15.2	27.4 ± 22.2	33.1 ± 16.4
Pharmaceutical drugs	15	19.0	4.5 ± 2.1	26.2 ± 37.4	1.7 ± 0.8	13.0 ± 13.5	20.6 ± 22.6	36.8 ± 13.5
Renal failure	3	3.8	3.4 ± 2.8	16.8 ± 10.7	2.5 ± 1.6	10.7 ± 9.0	17.0 ± 15.8	28.0 ± 7.0
Hyperprolactinemia	3	3.8	2.9 ± 0.6	104.8 ± 131.2	2.1 ± 0.6	4.9 ± 5.4	17.2 ± 26.6	15.5 ± 13.1
Liver chronic disease	2	2.5	4.9 ± 2.2	12.5 ± 5.4	1.6 ± 0.5	4.6 ± 0.4	6.3 ± 1.5	57.0 ± 29.7
Hyperthyroidism	2	2.5	3.3 ± 0.2	6.5 ± 2.1	0.01 ± 0.01	10.2 ± 1.7	7.9 ± 1.2	27.0 ± 2.8
Anabolic steroids	2	2.5	3.8 ± 0.1	8.8 ± 1.1	1.8 ± 0.2	4.2 ± 3.3	6.0 ± 0.9	29.1 ± 4.2
Adrenal carcinoma	1	1.3	2.4					78.1
Persistent puberty	1	1.3	2.3	2.8	0.9	1.2	3.8	20

Idiopathic	34	43.0	4.3 ± 1.0	11.8 ± 5.4	2.1 ± 1.6	5.1 ± 2.8	9.9 ± 8.3	26.8 ± 8.0

## Supplementary Materials

Supplementary Materials Methods used for hormone determinations are described.
