# Supplementary material for: Clinical and Etiological Aspects of Gynecomastia in Adult Males: A Multicenter Study
Source: Biomed Res Int. 2018 May 29;2018:8364824. doi: 10.1155/2018/8364824 (PMC5996435; doi:10.1155/2018/8364824)
Supplement: Supplementary Materials — Methods used for hormone determinations are described. [file 8364824.f1.pdf]

Supplementary Information

Methods used for hormone determinations: hormone levels were measured using the assay available at each of the participating sites, as described below:

- Total Testosterone (TT) was measured using:

1) Chemiluminescence on the Immulite 2000 (Siemens) analyzer, laboratory reference ranges: 3.0-8.8 ng/mL for adult males; the intra-assay coefficient of variation was 7.5% and the inter-assay coefficient of variation was 8.1% (n=145).

2) Chemiluminescence immunoassay on the Access Beckman Coulter analyzer, laboratory reference ranges: 3.0-9.0 ng/mL for adult males; the intra-assay coefficient variation was 10.0% and the inter-assay coefficient of variation was 8.5% (n=48).

3) Chemiluminescence immunoassay on the Cobas e411 (Roche) analyzer, laboratory reference ranges: 3.0-9.0 ng/ml for adult males; the intra-assay coefficient of variation was 1.8% and the inter-assay coefficient of variation was 7.7% (n=44)

- Prolactin was measured using:

1) Chemiluminescence immunoassay on the Architect (Abbott) analyzer, the lower limit of detection was 0.6 ng/mL, laboratory reference ranges: 5-20 ng/mL; the intra-assay coefficient of variation was 4.7% and the inter-assay coefficient of variation was 5.9% (n=145).

2) Chemiluminescence immunoassay Liasion Día Sorin, laboratory reference ranges: 2-14 ng/mL; the intra-assay coefficient of variation was 3.2% and the inter-assay coefficient of variation was 3.0% (n=48).

3) Chemiluminescence immunoassay on the ADVIA Centaur XP (Siemens) analyzer, laboratory reference ranges: 2-18 ng/mL; the intra-assay coefficient of variation was 2.8% and the inter-assay coefficient of variation was 6.3% (n=44).

- TSH was measured using:

1) Chemiluminescence immunoassay on the Architect (Abbott) analyzer, laboratory reference ranges: 0.47-4.64 ng/mL; the intra-assay coefficient of variation was 2.6% and the inter-assay coefficient of variation was 4.2% (n=145).

2) Chemiluminescence immunoassay on the Access Beckman Coulter analyzer, laboratory reference ranges: 0.5-4.5 uIU/mL; the intra-assay coefficient of variation was 5.6% and the inter-assay coefficient of variation was 3.02% (n=48).

3) Chemiluminescence immunoassay on the ADVIA Centaur XP (Siemens) analyzer, laboratory reference ranges: 0.35-4.5 mIU/L; the intra-assay coefficient of variation was 2.85% and the inter-assay coefficient of variation was 5.3% (n=44).

- LH was measured using:

1) Chemiluminescence immunoassay on the Architect (Abbott) analyzer, laboratory reference ranges: 2-12 mIU/mL; the intra-assay coefficient of variation was 4.1% and the inter-assay coefficient of variation was 4.3% (n=145).

2) Chemiluminescence immunoassay on the Access Beckman Coulter analyzer, laboratory reference ranges: 1.7-8.6 mIU/mL; the intra-assay coefficient of variation was 4.06% and the inter-assay coefficient of variation was 3.7% (n=48).

3) Chemiluminescence immunoassay on the ADVIA Centaur XP (Siemens) analyzer, laboratory reference ranges: 1.5-9.2 mIU/mL; the intra-assay coefficient of variation was 3.0% and the inter-assay coefficient of variation was 7.2% (n=44).

- FSH was measured using:

1) Chemiluminescence immunoassay on the Architect (Abbott) analyzer, laboratory reference ranges: 1-8 mIU/mL; the intra-assay coefficient of variation was 4.6% and the inter-assay coefficient of variation was 4.3% (n=145).

2) Chemiluminescence immunoassay on the Access Beckman Coulter analyzer, laboratory reference ranges: 1.5-12.4 mIU/mL; the intra-assay coefficient of variation was 4.06% and the inter-assay coefficient of variation was 3.7% (n=48).

3) Chemiluminescence immunoassay on the ADVIA Centaur XP (Siemens) analyzer, laboratory reference ranges: 1.0-14.0 mIU/mL; the intra-assay coefficient of variation was 2.9% and the inter-assay coefficient of variation was 6.2% (n=44).

- E2 was measured using:

1) Chemiluminescence immunoassay on the Architect (Abbott) analyzer, laboratory reference ranges: 18-44 pg/mL; the intra-assay coefficient of variation was 7.4% and the inter-assay coefficient of variation was 4.5% (n=145).

2) Chemiluminescence immunoassay on the Access Beckman Coulter analyzer, laboratory reference ranges: 10-60 pg/mL; the intra-assay coefficient of variation was 20% and the inter-assay coefficient of variation was 15% (n=48).

3) Electrochemiluminescence on the Cobas e411 (Roche) analyzer, laboratory reference ranges: 25-60 pg/mL; the intra-assay coefficient of variation was 3.5% and the inter-assay coefficient of variation was 7.7% (n=44).
